# Meta4P: A User-Friendly Tool to Parse Label-Free Quantitative Metaproteomic Data and Taxonomic/Functional Annotations

**DOI:** 10.1021/acs.jproteome.2c00803

**Published:** 2023-04-28

**Authors:** Massimo Porcheddu, Marcello Abbondio, Laura De Diego, Sergio Uzzau, Alessandro Tanca

**Affiliations:** † Department of Biomedical Sciences, University of Sassari, Viale San Pietro 43/B, 07100 Sassari, Italy

**Keywords:** bioinformatics, proteomic data analysis, functional annotation, label-free quantification, metaproteomics, spectral counting, taxonomic annotation

## Abstract

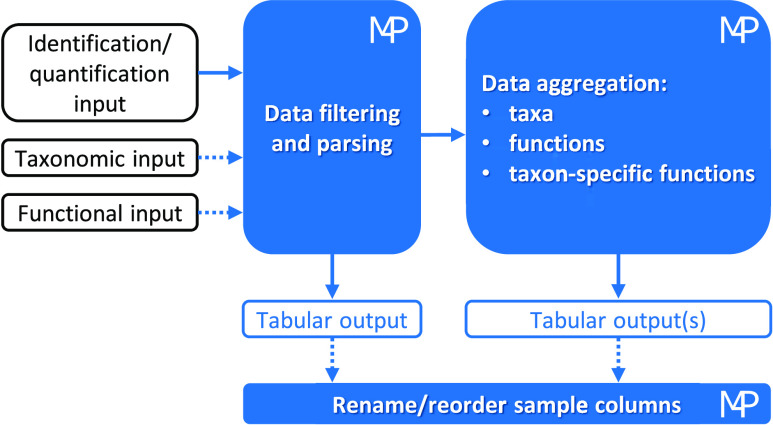

We present Meta4P (MetaProteins-Peptides-PSMs Parser), an easy-to-use bioinformatic application designed to integrate label-free quantitative metaproteomic data with taxonomic and functional annotations. Meta4P can retrieve, filter, and process identification and quantification data from three levels of inputs (proteins, peptides, PSMs) in different file formats. Abundance data can be combined with taxonomic and functional information and aggregated at different and customizable levels, including taxon-specific functions and pathways. Meta4P output tables, available in various formats, are ready to be used as inputs for downstream statistical analyses. This user-friendly tool is expected to provide a useful contribution to the field of metaproteomic data analysis, helping make it more manageable and straightforward.

## Introduction

Metaproteomics enables the functional characterization of microbial communities, adding valuable information to the expanding field of microbiome science.^1,2^ Several key challenges exist in metaproteomic data analysis, mainly depending on sample complexity. For instance, searches against large databases are usually required, as biological samples analyzed in metaproteomic studies often contain a large number of different species. In addition, metaproteomic data processing poses numerous redundancy (high sequence homology between proteins from different taxa) and annotation (many understudied taxa and/or uncharacterized proteins) issues.

Several bioinformatic platforms specifically designed for metaproteomic data analysis (thus aiming at tackling its related issues) have been made available in the last years, with a few of them also providing a graphical user interface (GUI).^3−8^ These tools embed open source/noncommercial search engines and enable parallel or sequential database searches using multiple engines. As an alternative, the combination of Sequest-HT and Percolator, available in the Proteome Discoverer (PD) platform from Thermo Fisher Scientific, can reach high identification yields even when using large databases and a single-search strategy.^9^

Among label-free quantification approaches used in proteomics, MS1-based quantification enables more accurate quantitative measurements as compared to spectral counting.^10^ In this respect, MaxQuant-based tools are suited to perform MS1-based quantification and to apply this strategy to multiple samples thanks to the “match-between-runs” function.^11^ The “Minora Feature Detector”, “Feature mapper”, and “Precursor Ions Quantifier” nodes available in PD can as well carry out MS1-based quantification and LC/MS peak alignment, providing two types of quantitative measures (peak intensity or area). Based on a recent report, PD’s Minora can outperform MaxQuant in terms of quantification yield and dynamic range.^12^ These types of abundance data can be provided in different formats, depending on the software used; standard formats for proteomics quantitative data exchange, such as mzTab, are also available.^13^

Although directly retrieving taxonomic annotations assigned to the (whole) protein sequences included in the database is an option, annotating the tryptic peptide sequences actually identified by mass spectrometry using a lowest common ancestor strategy (previously developed for metagenomic data)^14^ can provide more specific and robust results.^15^ The Unipept tool series allow metaproteome researchers to apply this latter approach for efficient taxonomic annotation of metaproteomes.^16−18^

Among the numerous computational solutions enabling functional annotation of protein sequences,^19,20^ the eggNOG-mapper web application offers the opportunity to straightforwardly analyze protein fasta files and to directly retrieve a wide range of functional information,^21,22^ including the well-curated Kyoto Encyclopedia of Genes and Genomes (KEGG) orthology, pathway, module, and reaction annotations.^23^

Moreover, a few further attempts have been made to develop bioinformatic applications able to integrate identification, quantification, and annotation outputs from different sources and to connect them with downstream statistical analysis.^24−26^ Among them, Prophane is compatible with many different input formats and offers various functional annotation strategies, but it cannot manage MS1-based quantification data. MetaQuantome can parse MS1-based quantification data, but it shows flexibility limitations in terms of input/output file formats and range of functional annotations available. Furthermore, to our knowledge, none of these tools are currently downloadable as stand-alone applications.

Here we present Meta4P (MetaProteins-Peptides-PSMs Parser), a user-friendly GUI tool aimed to allow researchers, even those with limited experience in informatics, to parse and integrate large label-free quantitative metaproteomic data sets with taxonomic and functional annotations. While Meta4P can directly handle files exported from PD, Unipept, and eggNOG-mapper, it also shows a high degree of flexibility, being compatible with different input levels (proteins, peptides, PSMs), file formats (including mzTab), and label-free quantitative measurements (based on MS1 peak intensity/area or PSM counts). The tool has been designed to aggregate abundance data based on taxonomic, functional, and/or taxon-specific functional annotations, thus allowing the user to manage and export a wide range of quantitative biological information.

## IMPLEMENTATION

### Meta4P Development

Meta4P executable files (for Windows and MacOS), along with a user guide and input/output template files, can be downloaded at https://github.com/TheMassimo/Meta4P/releases, while the source code is freely available for download at https://github.com/TheMassimo/Meta4P. The software is released as a multimodule Python code and was created using Python version 3.10.5. It features tools for processing mass spectrometry identification and quantification data, as well as taxonomic/functional annotation data. Meta4P makes extensive use of other open-source libraries including Tkinter for the interface, pandas and NumPy for scientific and numerical computing and Thread for multithreading functions. Meta4P is designed to receive as input the files containing the results of the identification/quantification and annotation analyses, subsequently allowing the user to (i) extrapolate the identification and quantification data of interest from PD, mzTab and generic tabular files; (ii) cross them with annotation data (specifically exported from the web applications Unipept and eggNOG-mapper or in a generic tabular format); (iii) retrieve specific functional annotation information from the KEGG online database; (iv) perform multiple data processing and aggregation operations to generate tabular output files useful for downstream statistical analysis; (v) rename and/or reorder sample column headers in the final output.

### Meta4P Workflow

**Figure 1:**
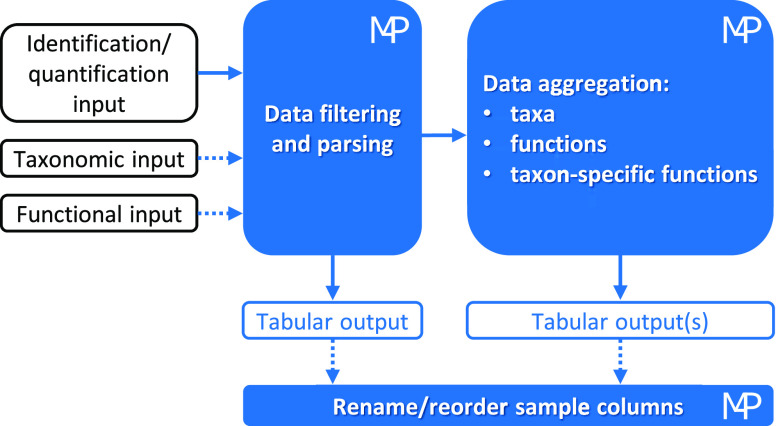
Schematic diagram of the Meta4P workflow. The first (mandatory) input can be a PD export file (in xlsx or txt format), a standard mzTab file, or a generic tabular file (in xlsx, txt, or other tab-separated format) and contains identification and quantification data (proteins with MS1-based intensity/area values, peptides with MS1-based intensity/area values or PSMs). The second and third (optional) inputs provide taxonomic and functional annotations, respectively; these can come from specific Unipept and eggNOG-mapper outputs, respectively, or from generic tabular files. Then, Meta4P integrates all data and aggregates them based on taxonomic, functional, and/or taxon-specific functional annotations by summing for each sample the abundances of all features (proteins or peptides) sharing the same annotation. At any stage, tabular outputs can be exported in xlsx, txt, or generic tab-separated format. Finally, when necessary, users can customize header and order of sample columns in one or more of the previously exported output tables.

A schematic illustration of the Meta4P workflow is provided in Figure 1.

Meta4P can manage three levels of inputs corresponding to three different label-free metaproteomic analysis approaches: proteins (protein identifications with MS1-based abundances), peptides (peptide identifications with MS1-based abundances), and PSMs (peptide identifications with PSM abundances). These data can be retrieved from PD export files (in xlsx or txt format), a standard mzTab file, or generic tabular files (in xlsx, txt or another tab-separated format). Once the input file is uploaded, Meta4P allows the user to (re)normalize abundances after filtering, to replace missing values with zeros, and/or to filter data based on the number/percentage of valid values; specifically, PD files can also be filtered according to different information related to protein/peptide grouping, quantification, statistical confidence, and protein/peptide/database description. When starting from a PSMs input, the software calculates for each peptide the number of PSMs detected per sample and reports the obtained values next to the corresponding peptide sequence.

Then, the user can upload a taxonomic and/or functional annotation input, if available. Unipept standard tab-separated outputs (including the following levels: LCA, superkingdom, phylum, class, order, family, genus, species) and eggNOG-mapper standard outputs (including the following levels: COG category, GO category, EC number, CAZy code, as well as KEGG KO, Pathway, Module, and Reaction annotations; multiple annotations per level are allowed) can be used as taxonomic and functional Meta4P inputs, respectively. Alternatively, in both cases a generic tab-separated input can also be uploaded. When dealing with peptide/PSM quantitative data, the user can choose between protein- and peptide-based functional annotations; in the former case, the tool associates each identified peptide sequence with the annotation of the master protein(s) (i.e., the representative sequence within a group of proteins sharing one or more peptides^27^) it belongs to. In addition, an option allows the user to keep missing annotations as empty cells or to denominate them as “unassigned”; only in the latter case the protein/peptide abundances related to missing annotations are considered in the following data aggregation step. As a further option, the user can retrieve and include the annotation names provided by the KEGG database for all KEGG annotation categories. Once the annotation files are uploaded, taxonomic and/or functional data are parsed and associated with the previously retrieved identification and quantification data. At any stage, an output table (in xlsx, txt, or generic tab-separated format) containing all the selected information can be exported by the user.

**Figure 2:**
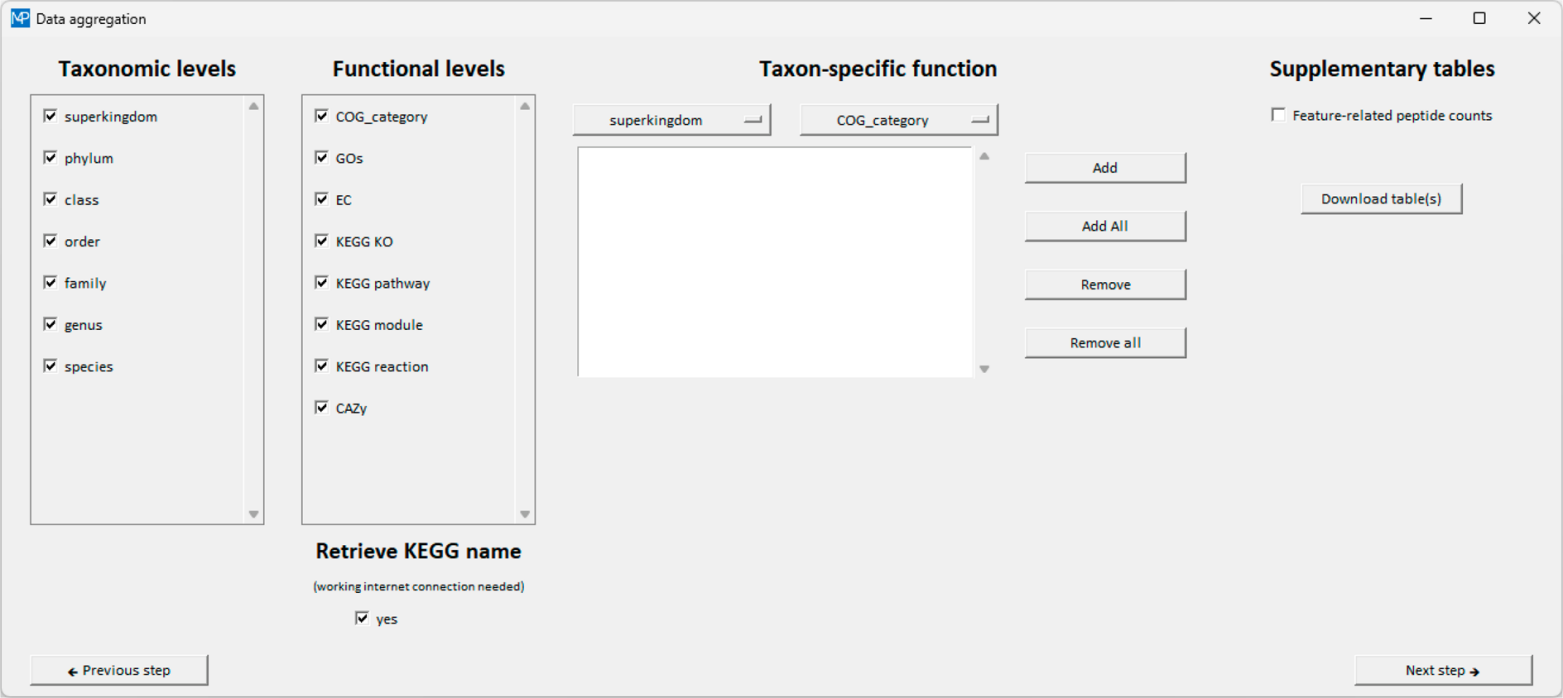
Meta4P “Data aggregation” window. The user can select specific taxonomic (left) and/or functional (middle-left) levels among those contained in the respective input files. Moreover, it is possible to generate taxon-specific functions (middle-right) by combining taxonomic and functional annotations (each with its drop-down menu). Clicking on “Download table(s)” the user can export an output tabular file (in xlsx, txt, or generic tab-separated format) for each of the selected categories, containing one feature per row along with its aggregated (summed) abundance values measured in each sample. The user can also choose to retrieve the name corresponding to each KEGG annotation number and/or to generate supplementary table(s) reporting the number of proteins/peptides that contributed to the (summed) abundances listed in the main output table(s).

At this point, quantitative data can be aggregated based on taxonomic, functional, and/or taxon-specific functional annotations (the Meta4P window dedicated to this operation is shown in Figure 2). In other words, the abundances of all proteins/peptides sharing the same annotation are summed for each sample. Taxon-specific functions can be customized by combining a taxonomic level with a functional level (from their respective drop-down menus), according to all possible combinations. At the end, the list(s) of all annotation features (taxa, functions, and/or taxon-specific functions) detected in the data set and belonging to the selected annotation level(s), together with their aggregated abundance values, can be downloaded as tabular file(s) (xlsx, txt, or generic tab-separated format format). Again, the user can also retrieve and include in the table(s) the annotation names provided by the KEGG database for all the selected KEGG categories. An additional table (for each output table selected) can be downloaded containing the feature-related protein/peptide counts, i.e., the number of proteins/peptides per sample for which an abundance value was measured and that, therefore, contributed to the summed abundance showed as aggregated value in the main table.

Finally, Meta4P allows users to customize name and order of sample columns in one or more of the output tables. The final tables might then be directly used as inputs for downstream statistical analyses and graph generation, which can be performed using dedicated computational platforms (e.g., Perseus^28^).

## APPLICATION EXAMPLE

To apply Meta4P to a real-world metaproteomic data set, we downloaded a PD pdresult file deposited in PRIDE (data set identifier PXD017467) from a study analyzing colonic luminal content samples collected from 24 colon cancer patients.^29^ The pdresult file was opened in PD (version 2.5.0.400) to export a “Peptide Groups” table, containing a list of peptide sequences along with related identification and quantification data (Data set S1; 57,102 entries). A fasta file was also exported, containing all proteins designed as “Master Proteins” upon protein grouping (according to a specific filter applied to the “Proteins” table). Peptide MS1-based quantitative data (namely, peak intensities normalized according to total peptide amount, as set in the PD “Precursor Ions Quantifier” node) were used as abundance measures. The peptide sequences identified in the study were annotated taxonomically using Unipept Desktop (version 2.0.0);^18^ all available options (“equate I and L”, “filter duplicate peptides”, and “advanced missed cleavage handling”) were checked and a tab-separated (although provided in csv format) output file was downloaded (Data set S2). In parallel, the fasta file was subjected to functional annotation using the eggNOG-mapper web application (http://eggnog-mapper.embl.de, version 2.1.9;^22^Data set S3).

In this application example, we exploited Meta4P to parse the identification, quantification, and annotation data contained in the three above-mentioned files (Data set S1, S2, and S3), with the final aim of identifying microbial family specific metabolic pathways actively working in the colonic microbiome of the studied patients. To obtain this information, we downloaded the Meta4P executable file on a Windows computer (processor Intel Core i7-5500U CPU @ 2.40 GHz 2.40 GHz, 8GB RAM), started a new analysis and then selected sequentially “Proteome Discoverer export” as input file type and “Peptides” as data level, according to the characteristics of the input file available. In the “Peptides” window, we uploaded Data set S1 as Excel input file (upload time: about 1 min). Among filters/options, we selected the following: “High” in the “Confidence” menu; “Shared” in the “Quan info” box (to keep all peptides with a valid abundance value); “MGDBinhouse”, “MGDBpublic”, and “MGDBinhouse;MGDBpublic” (with MGDB standing for metagenomic database) in the “Marker” box (to focus on microbial peptides); “(Re)normalize abundances after filtering” in the “Options” menu (to normalize the peptide abundances based on the summed abundance of all filter-passing peptides in each sample). Going on to the “Taxonomic menu” and “Taxonomic annotation” windows, we selected “Unipept output” and uploaded Data set S2) as “generic tab-separated values” input file (upload time: a few seconds). Then, in the “Functional menu” and “Functional annotation” windows, we selected “Protein functional input” (as functional annotation had been performed on a protein fasta file), “eggNOG-mapper output”, and uploaded Data set S3 as Excel input file (upload time: about 10 s). Finally, in the “Data aggregation” window, we specifically selected the output we were interested in, i.e., family specific pathways. To this aim, within the “Taxon-specific function” section, we selected “family” in the left drop-down menu and “KEGG pathway” in the right drop-down menu, then clicking on the “Add” button. In addition, we selected “yes” in the “Retrieve KEGG name” option, to retrieve the full name of each pathway and list it beside the KEGG pathway code in the output table. Upon clicking on “Download table(s)” and leaving the “prefix/suffix” boxes blank, we were able to download the output tabular file in txt format (upload time: a few seconds). The resulting file, available as Supporting Information (Data set S4), contains the list of the microbial family specific pathways detected in the samples (with taxonomic and functional information provided in three adjacent columns, having “family”, “KEGG_Pathway”, and “Pathway name” as headers), along with their abundance values measured in each sample.

## Conclusion

Meta4P is an easy-to-use bioinformatic application enabling metaproteomics researchers to parse, integrate, and aggregate peptide/protein identification, quantification, and taxonomic/functional annotation data. The final Meta4P outputs (i.e., the abundance profiles of all taxa, functions, and taxon-specific functions detected in the data set of interest) are tabular files available in different formats, directly ready for downstream statistical analyses. We anticipate that this user-friendly tool might contribute to make metaproteomic data analysis more accessible and standardized.

## Supporting Information Available

Data set S1: “Peptide Groups” table (containing peptide sequences, along with identification and quantification data, as exported from Proteome Discoverer) based on the pdresult file downloaded from PRIDE data set PXD017467 (XLSX)
Data set S2: Unipept annotation output obtained from the peptide sequences identified in data set PXD017467 (ZIP)
Data set S3: Output of eggNOG-mapper functional annotation of the Master Protein sequences identified in data set PXD017467 (XLSX)
Data set S4: Abundances of family specific KEGG pathways in each sample, as obtained from Meta4P analysis of Data sets S1, S2, and S3 (TXT)
